# Meibum Lipidomic Analysis in Evaporative Dry Eye Subjects

**DOI:** 10.3390/ijms25094782

**Published:** 2024-04-27

**Authors:** Jacobo Garcia-Queiruga, Hugo Pena-Verdeal, Belen Sabucedo-Villamarin, Monica Paz-Tarrio, Esteban Guitian-Fernandez, Carlos Garcia-Resua, Eva Yebra-Pimentel, Maria J. Giraldez

**Affiliations:** 1 GI-2092 Optometry, Departamento de Física Aplicada, Facultad de Óptica y Optometría, Universidade de Santiago de Compostela, Campus Vida s/n, 15701 Santiago de Compostela, Spain; jacobogarcia.queiruga@usc.es (J.G.-Q.); hugo.pena.verdeal@usc.es (H.P.-V.); belen.sabucedo@rai.usc.es (B.S.-V.); carlos.garcia.resua@usc.es (C.G.-R.); eva.yebra-pimentel@usc.es (E.Y.-P.); 2 AC-24 Optometry, Instituto de Investigación Sanitaria de Santiago de Compostela (IDIS), Travesía da Choupana, 15701 Santiago de Compostela, Spain; 3 Mass Spectrometry and Proteomic Unit, Área de Infraestruturas de Investigación, Universidade de Santiago de Compostela, Campus Vida s/n, 15701 Santiago de Compostela, Spain; monica.paz@usc.es (M.P.-T.); esteban.guitian@usc.es (E.G.-F.)

**Keywords:** meibum, tear film lipid layer, eyelid margin abnormalities, meibomian gland, lipid layer pattern

## Abstract

Meibomian Glands (MG) are sebaceous glands responsible for the production of meibum, the main component of the Tear Film Lipid Layer (TFLL). The TFLL facilitates the spread of the tear film over the ocular surface, provides stability and reduces tear evaporation. Alterations in meibum composition lead to different ocular alterations like Meibomian Gland Dysfunction (MGD) and subsequent Evaporative Dry Eye (EDE). The aim of the present study was to investigate the composition and abundance of meibum lipids and their relationship with eyelid margin abnormalities, lipid layer patterns and MG status. The study utilizes a lipidomic approach to identify and quantify lipids in meibum samples using an Elute UHPLC system. This system considered all four dimensions (mass/charge, retention time, ion mobility and intensity) to provide the accurate identification of lipid species. Samples were categorized as healthy or low/no signs of alteration (group 1) or severe signs of alteration or EDE/MGD (group 2). The current investigation found differences in Variable Importance in Projection lipid abundance between both groups for the MGD signs studied. Changes in meibum composition occur and are related to higher scores in eyelid margin hyperaemia, eyelid margin irregularity, MG orifice plugging, MG loss and lipid layer pattern.

## 1. Introduction

Meibomian Glands (MG) are sebaceous glands located in the tarsal plates of both superior and inferior eyelids, and which excrete into the eyelid margin where their orifices are located [1]. These glands are responsible for the production of the meibum, which is a lipid secretion and the main component of the Tear Film Lipid Layer (TFLL) [2,3]. Reducing the tear evaporation rate and reducing the surface tension to facilitate the spreading of the tear film on the ocular surface are the main functions of the TFLL, both of which are related to the non-polar and polar lipids that constitute it [3]. The tear film contains water, electrolytes, mucins and a large compound of proteins and lipids, forming a bilayer made of a hydrated mucus layer and a lipid layer [4]. The TFLL is a biphase of non-polar and polar lipids, each with particular characteristics that allow interaction with air (non-polar lipids) and with the muco-aqueous layer (polar lipids) [5]. Changes in the chemical composition of any of the layers can alter the structure, function, or dynamics of the tear film, disrupting the homeostasis of the ocular surface, which may result in dry eye disease (DED) [6,7,8,9].

Meibomian Gland Dysfunction (MGD) is the primary cause of Evaporative Dry Eye (EDE), which is the most prevalent type of DED in the entire global population [10]. The diagnosis of EDE due to MGD include a range of clinical tests to identify the presence of symptoms and different signs compatible with eyelid margin abnormalities, alterations in the MG morphology and anormal tear film dynamics [11,12,13,14,15]. These clinical tests evaluate various ocular characteristics of different ocular structures. The features regarding eyelid margin abnormalities include eyelid margin hyperaemia, irregularity, thickening and plugging of the MG orifices [12]. In the case of MG morphology, meibography images captured with infrared cameras allow clinicians to evaluate the MG loss, MG drop out and partial glands [12,13]. Measuring the thickness of the TFLL provides indirect information about the status of the MG secretion, so clinicians analyse Lipid Layer Patterns (LLP) with interferometers to assess it [14,15]. Even though MGD can be present in different forms (highly productive MGD or obstructive MGD), the most prevalent is the obstructive, which is related to the hyper-keratinisation of the MG main duct, the plugging of the MG orifices in the eyelid margin and the destruction of the MGs [1,2]. The obstruction of the MG affects the quantity and quality of the meibum, where thicker secretions are more difficult to spread over the ocular surface, leading to tear film instability [3,4,16]. 

The meibum is a lipid-rich secretion mainly composed of non-polar lipids such as wax esters, cholesterol esters (CE), triacylglycerols (TG) and polar lipids such as ceramides (Cer), sphingomyelins and phospholipids, among others [2]. The lipidomic profile of the meibum has been studied by performing different techniques in animal and human models, such as thin layer chromatography, gas chromatography, and high-performance liquid chromatography [5,17,18,19,20,21]. Different researchers have found changes in the meibum composition in various states of ocular diseases such as DED and MGD [7,22,23]. It has been observed that changes in meibum composition are not only due to DED, but also to other comorbidities such as diabetes mellitus [21]. In the same way that other ocular physiological aspects such as cataracts or eyelid ptosis are influenced by ageing, changes in the composition of the meibum have also been correlated with older subjects [9]. In addition, researchers have identified a significant negative correlation between the abundance of non-polar lipids and the presence of specific symptoms, including ocular fatigue, blurred vision and decreased visual acuity, among MGD subjects [9]. Specific information on which lipids influence the manifestation of each alteration in the multiple eyelid margin abnormalities, MG features or TFLL has not been found in depth. Paranjpe et al. [7] studied some of these ocular sings of alteration related to different aspects of the MGs, such as MG orifice plugging and MG atrophy or loss. They found an association between these MGD signs and changes in meibum composition, with an increase in sphingomieline and a drop in Cer [7]. In another report, cholesterol esters and wax esters showed no relationship between MGD severity status and precorneal tear film thinning [24]. 

In response to the demands of researchers in the MGs and meibum field for further research to elucidate the pathophysiology of MGD, the aim of the present study was to determine which lipids are present in high amounts in the different characteristics that should be studied for the proper diagnosis of MGD and the subsequent DED (eyelid margin abnormalities, LLP, and morphological changes in the MG). Also, the lipid identification method performed in the present study is a novel four-dimensional workflow that considered all four dimensions (mass/charge [*m*/*z*], retention time, ion mobility and intensity) to provide the most accurate lipid species identification.

## 2. Results

### 2.1. Lipidomic Profiling Untargeted Analysis

A total of 44 meibum samples from upper and lower eyelids were analysed using PASEF^®^ MS/MS mode (Online Parallel Accumulation-Serial Fragmentation) for the untargeted analysis [25]. A total of 131 lipids were identified in the data analysis. Among these, 38 lipids were determined in at least 95% of the samples. The number of lipids identified according to their lipid class is listed in Table 1.

### 2.2. Comparison of Meibum Lipids between Groups

**Figure 1:**
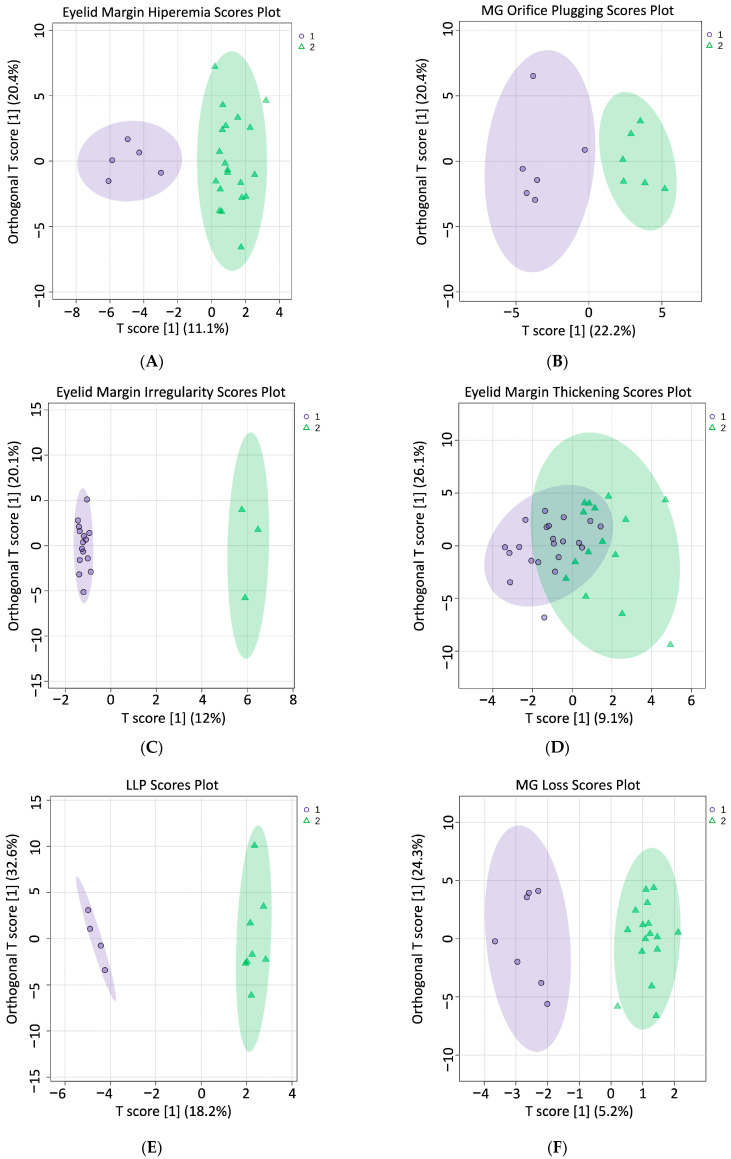

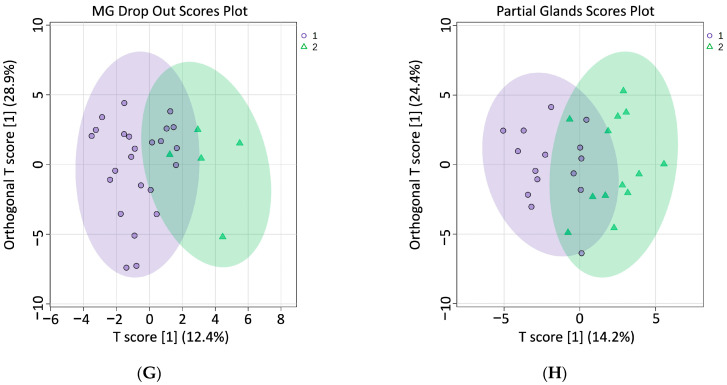
Ortho-DA score plots for eyelid margin abnormalities, LLPs and meibography. (**A**) Hyperaemia ortho-DA; (**B**) MG orifice plugging ortho-DA; (**C**) irregularity ortho-DA; (**D**) eyelid margin thickening ortho-DA; (**E**) LLP ortho-DA; (**F**) MG loss ortho-DA; (**G**) MG drop out ortho-DA; (**H**) partial glands ortho-DA. Score plots are shown for “T score” and “Orthogonal T score.” Purple circles and green triangles represents samples from group 1 and group 2, respectively. LLP: lipid layer pattern; MG: meibomian gland; Ortho-DA: supervised orthogonal partial least squares discriminant analysis.

A supervised orthogonal partial least squares discriminant analysis (ortho-DA) was performed to discriminate between groups 1 (healthy subjects with no or low levels of alteration in the studied parameters) and 2 (EDE/MGD subjects with severe signs of alteration in the studied parameters) (represented in Figure 1 as purple circles for group 1 and green triangles for group 2) and to graphically observe whether the species present in each group for each characteristic studied were different. The score plot of ortho-DA was performed on the different eyelid margin characteristics studied (Figure 1). Ortho-DA score plots completely distinguished groups 1 and 2 in eyelid margin hyperaemia (Figure 1A), MG orifice plugging (Figure 1B) and eyelid margin irregularity (Figure 1C). Nevertheless, the ortho-DA score plot showed an overlapping of the lipid species between groups for eyelid margin thickening (Figure 1D). A score plot of ortho-DA was also performed for LLP, MG loss, MG drop out and partial glands characteristics. Ortho-DA score plots completely distinguished groups 1 and 2 from the LLPs (Figure 1E) and MG loss (Figure 1F). Nevertheless, they showed an overlapping of lipid species between both groups on MG drop out (Figure 1G) and partial glands (Figure 1H).

**Figure 2:**
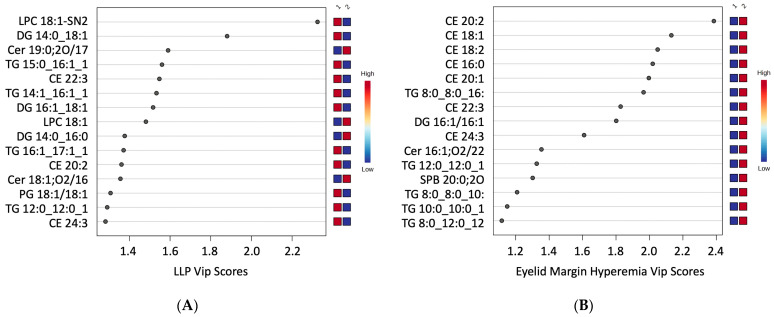

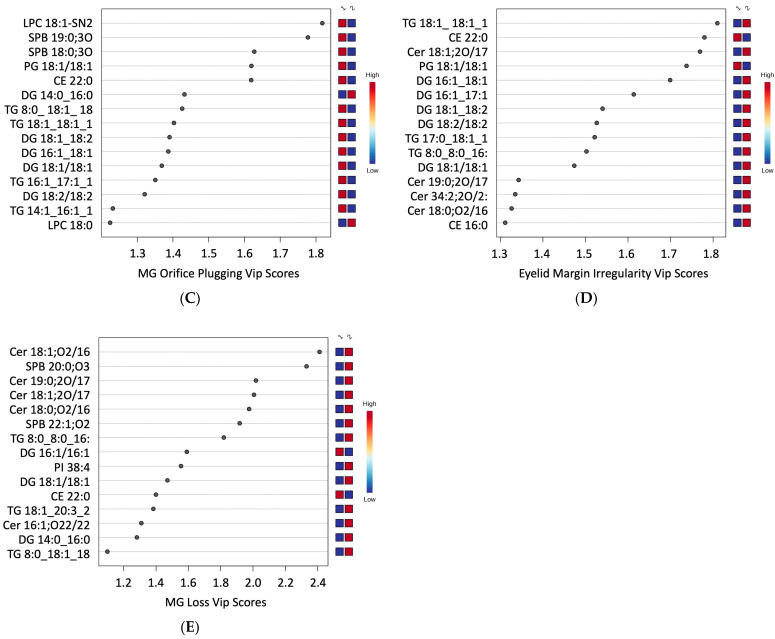
VIP scores of first discriminant function. VIP scores show the top lipid species selected by the ortho-DA analysis for component T. (**A**) VIP scores from LLP study. (**B**) VIP scores from eyelid margin hyperaemia study. (**C**) VIP scores from MG orifice plugging study. (**D**) VIP scores from eyelid margin irregularity study. (**E**) VIP scores from MG loss study. LLP: Lipid Layer Pattern; MG: Meibomian Gland; VIP: Variable Importance in Protection.

The lipids with Variable Importance in Projection (VIP) from the ortho-DA higher than 1.5 (Figure 2) were considered biologically relevant in the LLPs (Figure 2A), eyelid margin hyperaemia (Figure 2B), MG orifice plugging (Figure 2C), eyelid margin irregularity (Figure 2D) and MG loss features (Figure 2E).

**Figure 3:**
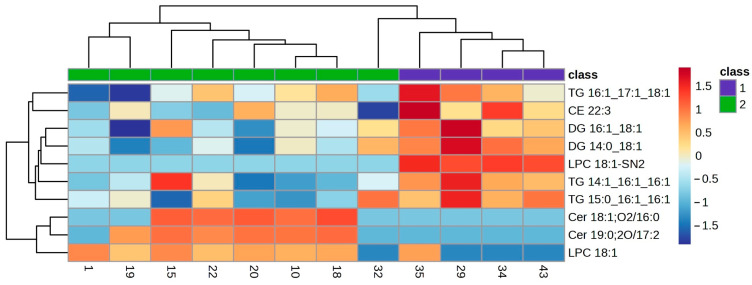
Clustering analysis. Hierarchical clustering analysis from LLP VIP lipids. Clustering analysis of 10 VIP lipid species from the LLP statistical analysis. Each column represents individual samples, and each row represents individual lipids. Blue and red colours represent low and high lipid abundance, respectively. Sample groups are represented by colours purple and green for groups 1 and 2, respectively. LLP: Lipid Layer Pattern; VIP: Variable Importance in Projection.

**Figure 4:**
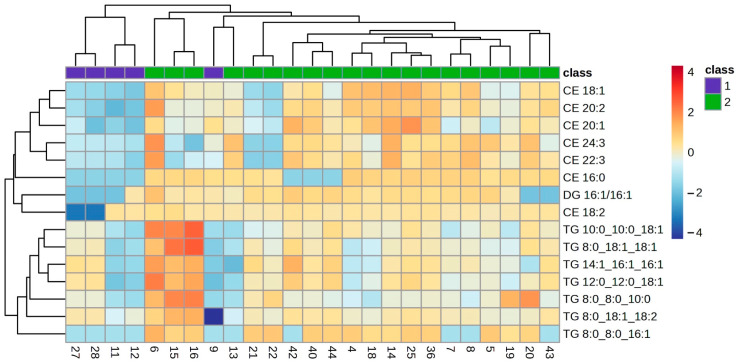
Clustering analysis. Hierarchical clustering analysis from eyelid margin hyperaemia VIP lipids. Clustering analysis of 15 VIP lipid species from the eyelid margin hyperaemia statistical analysis. Each column represents individual samples, and each row represents individual lipids. Blue and red colours represent low and high lipid abundance, respectively. Sample groups are represented by colours purple and green for groups 1 and 2, respectively. VIP: Variable Importance in Projection.

**Figure 5:**
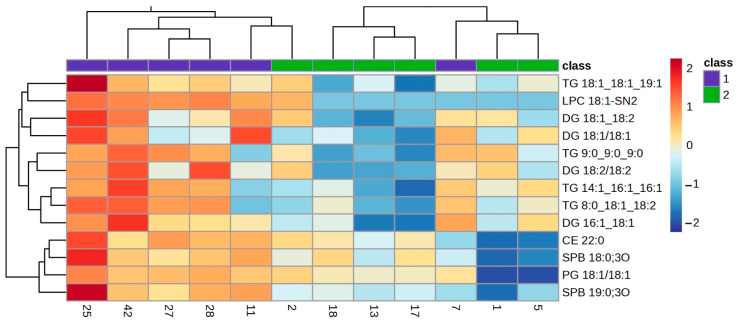
Clustering analysis. Hierarchical clustering analysis from MG orifice plugging VIP lipids. Clustering analysis of 13 VIP lipid species from the MG orifice plugging statistical analysis. Each column represents individual samples, and each row represents individual lipids. Blue and red colours represent low and high lipid abundance, respectively. Sample groups are represented by colours purple and green for groups 1 and 2, respectively. MG: Meibomian Gland; VIP: Variable Importance in Projection.

**Figure 6:**
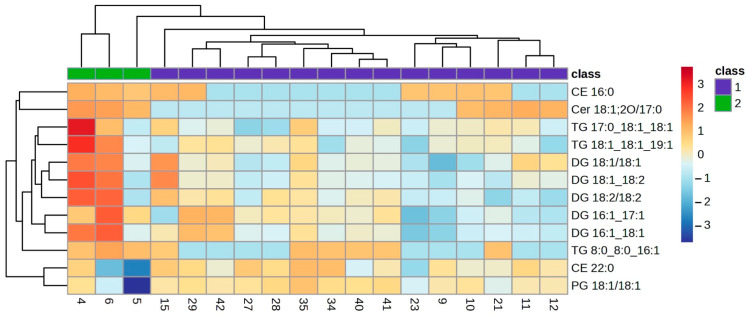
Clustering analysis. Hierarchical clustering analysis from eyelid margin irregularity VIP lipids. Clustering analysis of 12 VIP lipid species from the eyelid margin irregularity statistical analysis. Each column represents individual samples, and each row represents individual lipids. Blue and red colours represent low and high lipid abundance, respectively. Sample groups are represented by colours purple and green for groups 1 and 2, respectively. VIP: Variable Importance in Projection.

**Figure 7:**
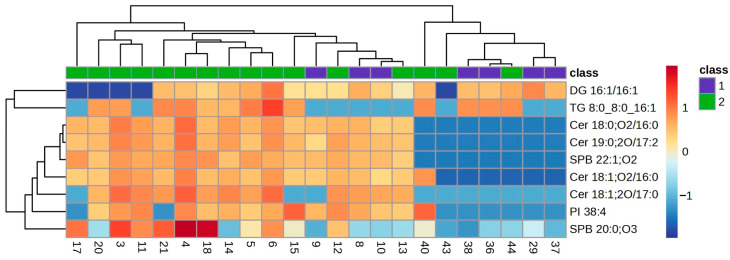
Clustering analysis. Hierarchical clustering analysis from MG loss VIP lipids. Clustering analysis of 9 VIP lipid species from the MG loss statistical analysis. Each column represents individual samples, and each row represents individual lipids. Blue and red colours represent low and high lipid abundance, respectively. Sample groups are represented by colours purple and green for groups 1 and 2, respectively. MG: Meibomian Gland; VIP: Variable Importance in Projection.

The lipid profile of the VIP lipids was studied in detail using a clustering analysis which was represented in a heatmap for each of the ocular features. Heatmaps of the clustering analysis for the different features analysed show graphically which VIP lipids are present or absent in each group (Figure 3, Figure 4, Figure 5, Figure 6 and Figure 7). Each heatmap shows the VIP lipids in rows and the samples of each eye (secretion collected from both eyelids) analysed in columns. The most similar samples are displayed closely. The first row of boxes represents the group to which samples correspond, with purple for group 1 (healthy subjects with no or low levels of alteration in the studied parameters) and green for group 2 (EDE/MGD subjects with severe signs of alteration in the studied parameters). The colour of each box indicates the high (red) or low (blue) abundance of each VIP lipid.

Heatmaps represent the VIP lipids whose abundance differs between samples according to the ortho-DA analysis. Heatmaps show the differences between group 1 (boxes under the first box purple row) and group 2 (boxes under the first box green row) graphically. While group 1 samples showed high or low abundance of each VIP lipid identified in each ocular parameter analysed (LLPs in Figure 3, eyelid margin hyperaemia in Figure 4, MG orifice plugging in Figure 5, eyelid margin irregularity in Figure 6, and MG loss in Figure 7), group 2 showed the opposite.

## 3. Discussion

The aim of the present study was to determine which lipids are present in high amounts in those subjects who showed no alteration in the different characteristics of the eyelid margin, of the LLP and of the MG (group 1) compared to those with high degrees of alteration in all studied features (group 2). Only lipids classified as VIP were studied because of their relative presence in the meibum samples and their biological importance [5,26]. Overall, the findings of this study add valuable information to the literature by relating the presence of each lipid and its possible relationship to the severity of ocular disorders.

A total of eight ocular surface features were analysed in the present study, and ortho-DA only found differences in meibum composition due to characteristic alterations in five of them (LLPs, eyelid margin hyperaemia, MG orifice plugging, eyelid margin irregularity and MG loss). The LLPs could be the most representative characteristic of the meibum on the ocular surface because this feature represents the density of the TFLL. The TFLL is a biphase composed of polar and non-polar lipids, and the present study found 64 polar lipids and 67 non-polar lipids in the 44 samples analysed. Among those 131 lipids identified in the meibum samples, 10 of them were characterized as VIP for the LLP. The heatmap (Figure 4) shows an enhancement in the abundance of polar lipids (Cer 18:1;02/16:0, Cer 19:0;2O/17:2 and LPC 18:1) on the samples that presented thinner LLPs (group 2). Additionally, a clear reduction in the abundance of non-polar lipids (CE, TG and DG) is represented in the heatmap (Figure 3). This finding supports the hypothesis stated by different researchers, where thinner TFLL are related to the reduction in meibum production due to MGD, and where a change in the meibum composition, showing an increase in polar lipids, occurs [8,9,27]. Additionally, these findings are in accordance with those supporting the important structural role of the Cer in TFLL, where an increase in concentration leads to an increase in the melting temperature of the meibum, causing the destabilization of the TFLL [16]. 

In the case of eyelid margin hyperaemia, 15 VIP lipids were identified in the meibum samples of both groups. All those VIP lipids were non-polar species (CE, DG and TG) that showed differences in the ortho-DA between both groups. These non-polar lipids showed a variation in their abundance, from being absent in group 1 to being quite marked in group 2 (Figure 4). This observation could be related to the physiology changes that take place in those MGs from the subjects of the group 2. Those MGs are suffering from altered meibocyte maturation, but they do not stop excreting even though partial hyperkeratinisation occurs in the MG duct [1]. However, a decrease in all VIP lipids analysed from the MG orifice plugging feature in group 1 and group 2 can be observed in the heatmap (Figure 5). Both margin hyperaemia and MG orifice plugging findings in the heatmaps could be interpreted from a clinical perspective, because it is common to find high values of eyelid margin hyperaemia and margin telangiectasia related to high values of MG orifice plugging [6,28]. The decrease in lipid abundance found in the analysed samples between both groups for MG orifice plugging could be explained by the physico-chemical properties of the plug because the plugs are composed of polar species such as diacylglycerols [6]. The present study was not intended to collect the plugs, as it would have been necessary to generate a large force which could cause the lid margin to be scraped off [29]. If the plugs had been included in the samples, an increase in polar lipids would have been observed. However, a lower abundance of the same VIP lipids was found and a high amount of polar lipids was not found because the meibum collected came from those MGs that excreted due to being not plugged.

Eyelid margin irregularity could be found in severe states of MGD and EDE and in elderly populations [30,31]. Due to the small sample analysed in the present study, only 3 meibum samples were from subjects that showed eyelid margin irregularity (group 2), against 15 samples of healthy ones. Nevertheless, ortho-DA was able to discriminate between the two groups in terms of meibum composition (Figure 1C). The samples of group 2 showed higher amounts of non-polar lipids, such as CE, TG and DG, and just Cer formed the group of polar lipids (Figure 6). Two explanations could be attributed to these observations. First, the higher abundance of non-polar lipids observed in those eyelid margins with irregularity could be related to the morphology of those altered eyelid margins that could function as a bigger reservoir. Secondly, Cer is related to destabilization of the TFLL, and so an unstable TFLL could lead to an inefficient tear film that could not be adequately spread over the ocular surface remaining in the eyelid margin reservoir.

The quantification of MG loss by meibography has rapidly become popular for the diagnosis of MGD, but other features of the MGs also play an important role in the pathophysiology of the disease, such as eyelid margin abnormalities [12,32,33,34]. The ortho-DA analysis performed in the present study was able to discriminate between group 1 and group 2 according to the presence of different lipids species and MG loss. Considerable amounts of SPB 22:1;O2 and SPB 20:0;O3 were found in samples of the group 1, but no sample of group 2 showed the presence of these polar lipids (Figure 7). An MG loss heatmap represented the VIP lipids present among the studied samples, and it showed a decrease in different lipid species for the group 2 samples, with particular emphasis on different Cer. The variation in lipid abundance observed in this study is directly linked to the alteration of the MG morphology. MGs that are reduced in size and number per eyelid produce less meibum compared to healthy and full-length MGs. Also, only digital force was applied during the collection of the meibum; no forceps were used to express the MGs, so meibum samples came primarily from the eyelid margin reservoir. Those subjects that presented severe MG loss had altered MGs, and the main ducts were at some point hyper-keratinised. The lower abundance of Cer shown in the heatmaps could be explained by the absence of a high force applied to force the excretion of the MGs, as their main ducts would be hyper-keratinised and could show high amounts of Cer due to its involvement in the keratinisation process [7,16].

The study of the lipid profile in extracts of complex lipids can be an arduous task that requires a lot of time to perform several types of analyses in database searches and for confident annotation. Lipids are a class of extraordinarily complex compounds, with a wide structural diversity. The work of performing such an annotation can be greatly minimized by applying a 4D workflow (*m*/*z* ratio, retention time, ion mobility and intensity). With this 4D workflow, the authors can speed up the annotation process of these lipids using data obtained by mass spectrometry and trapped ion mobility. By using PASEF^®^ [35] MS/MS mode (Online Parallel Accumulation-Serial Fragmentation), the authors could achieve greater coverage in the MS/MS fragmentations performed. Furthermore, by using mobility, the authors managed to clean the MS/MS spectra and improve the matching of the fragmentation rules or spectral libraries. Another third improvement factor is the acquired Collisional Cross Section (CCS) values, which can be used directly to increase confidence in the annotation of the lipids to be searched.

Regarding limitations, the present study used a cross-sectional design, which offers information only at a single time point, potentially overlooking longitudinal changes or causal relationships that may occur during the chronic process of the disease. Moreover, the manual expression technique used for collecting meibum samples may introduce variability in the quantity of samples collected. As other authors have demonstrated, forceps expression could be an alternative to obtain higher amounts of lipids [29], and also from those MG that have been obstructed. However, the use of forceps has the disadvantage that ocular anaesthesia must be applied due to the discomfort of the procedure. Finally, the small sample size, particularly concerning subjects with eyelid margin irregularity, restricts the generalizability of findings, as elderly individuals, potentially presenting with age-related comorbidities, were excluded from the study.

The findings of the present study have many clinical implications, such as offering valuable insights that could provide additional information to clinicians regarding the diagnosis and management of highly prevalent disorders such as MGD and EDE. This interdisciplinary collaboration between basic research and clinical application underscores the transformative power of scientific inquiry in advancing patient care and underscores the critical role of translational research in filling the gap between both areas. First, this investigation could enhance the implementation of meibum analysis by clinical laboratories of hospitals, as occurs with urine, faeces, mucus or blood samples. The implementation of this protocol in hospitals or eye care centres will have a significant clinical impact with a very low economic burden and will produce rapid analysis without being time-consuming. Understanding the correlation between meibum lipid composition and eye conditions will provide eye care professionals with more accurate strategies to treat these disorders and potentially improve the quality of life of affected patients. Second, the current findings have potential for transfer to the pharmaceutical industry, as novel tear substitutes could be developed and customized to each type of DED, focusing on the specific ocular alterations that show those patients suffering from EDE and/or MGD. DED management aims to alleviate ocular symptoms by reducing eye pain and discomfort. Identifying the specific lipids altered in each severity type of EDE/MGD is crucial as it could lead to targeted ocular therapies that restore ocular homeostasis, particularly significant in a chronic condition like DED. Presently, many tear substitutes and liposome sprays may not provide comprehensive relief for EDE/MGD, and in some cases, the compounds they contain might exacerbate patients’ conditions. Developing new treatments based on meibum findings holds promise for improving patient outcomes and benefiting pharmaceutical industries.

## 4. Materials and Methods

### 4.1. Sample and Study Design

The present cross-sectional study enrolled a total of 22 participants (44 eyes) with mean age 50.8 ± 14.5 years old (75% women). All the participants were recruited from the Optometry Clinic of the centre, and all signed a written consent form to be included in the study. The protocol adhered to the tenets of the Declaration of Helsinki and was approved by the Bioethics Committee of the institution (Approval Number: USC-40/2020). As the main inclusion criteria, participants could not have a history of eye diseases such as glaucoma, ocular allergy, age-related macular degeneration, ocular trauma or ocular surgery, nor have suffered from any systemic disease [36]. Participants were either healthy subjects or subjects previously diagnosed with EDE/MGD who were offered the opportunity to participate in the study.

In a single appointment, an ocular examination including LLP, eyelid margin exploration, meibography and meibum collection was performed.

### 4.2. Ocular Procedures

All ocular examinations were performed by the same observer, and all procedures were analysed by a second masked observer. The procedures were performed from least to most invasive to avoid any possible interaction between them [11]. The classification scheme followed for each procedure is summarized in Table 2.

#### 4.2.1. Lipid Layer Patterns

An EasyTear (Easytear S.R.L, Rovereto, Italy) interferometer was attached to the slit-lamp Topcon SL-D4 (Topcon Corporation, Tokyo, Japan) to observe LLPs [37]. A video of LLPs for each eye was captured by the Topcon DC-4 (Topcon Corporation, Tokyo, Japan) camera mounted on the slit-lamp. LLPs were first described by Guillon and the thickness of the TFLL could be estimated due to the interferometric pattern. Also, Guillon’s scheme was followed to categorize LLPs into 5 grades (from thickest to thinnest lipid layer) [14] (Table 2). 

#### 4.2.2. Eyelid Margin Abnormalities

Once LLPs had been videotaped, the examination of upper and lower eyelid margins was performed under a Topcon SL-D4 slit-lamp. A video of each eye was captured for its analysis following the Arita et al. [12] grading scale. Several aspects of eyelid margin alterations were assessed, such as hyperemia, MG orifice plugging, irregularity and margin thickening (Table 2).

#### 4.2.3. Meibography

Meibography images were taken with OCULUS Keratograph 5M (OCULUS Optikgeräte GmbH, Wetzlar, Germany), which was used to analyse the in vivo status of the MG. MG loss was calculated by subtracting the total area of the eyelid and the area of the MG [38]. The value obtained was expressed as a percentage, which was used to categorize each eyelid into the grades stated by Pult et al. [13] (Table 2). Also, the total number of partial MG and MG dropout results were classified following Arit et al. [12]’s scheme (Table 2).

#### 4.2.4. Meibum Sample Collection

**Figure 8:**
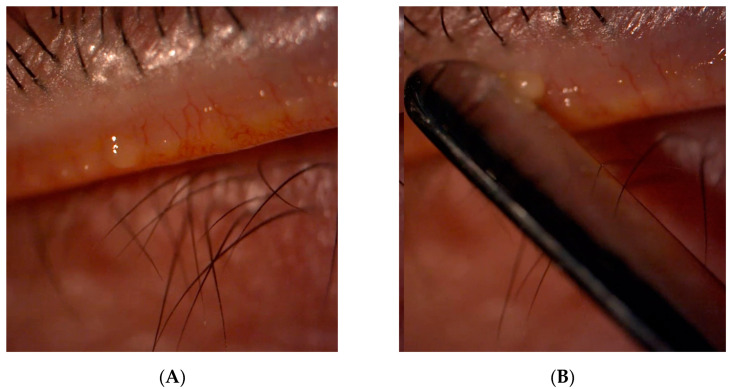
MG expression and meibum collection. (**A**) Meibum reaching the MG orifices at the eyelid margin; (**B**) sample collection with stainless steel spatula. MG: Meibomian Gland.

Participants were requested to position themselves properly in the chinrest of the slit-lamp and to maintain their gaze on the ceiling or to the floor, depending on which eyelid was being examined. A continuous pressure with the thumb for 30 s was applied to the lower and upper eyelids to force the MG to excrete the meibum and reach the MG orifice on the eyelid margin (Figure 8). A clean and sterilized stainless steel spatula was used to collect the meibum, first from the lower and then from the upper eyelid, taking care not scrape the eyelid margins [5]. The samples were placed in a clean topaz HPLC vial filled with 1 mL of chloroform. Each vial contained a sample collected from both lower and upper eyelids. Samples from both eyelids were analysed together because meibum secreted by both eyelids is responsible for tear film dynamics, and one sample can cloud the influence or alter the other due to meibum deposition at the eyelid margin. The samples were stored in a freezer at −30 °C until their later measurement.

### 4.3. Materials for Sample Preparation and Liquid Chromatography

Milli-Q water (Merk KGaA, Darmstadt, Germany), acetonitrile UHPLC-MS (Carlo Erba Reagents, Barcelona, Spain), propano-2-ol for LC/MS (Carlo Erba Reagents, Barcelona, Spain), chloroform HPLC grade (Fisher Chemical, Thermo Fisher Scientific, Waltham, MA, USA), ammonium formate for LCMS (Carlo Erba Reagents, Barcelona, Spain) and formic acid for LC-MS (Carlo Erba Reagents, Barcelona, Spain) were used in the present study.

### 4.4. Meibum Sample Preparation

Samples, once collected in topaz vials, were stored in a freezer at −30 °C until their later measurement. Before measuring them, they were evaporated for 30 min in a Speed-Vac Savant SPD121P-230 (Thermo Electron Corporation, Milford, MA, USA), and each sample was resuspended in a methanol–chloroform solution (1:1).

### 4.5. Liquid Chromatography–Mass Spectrometry Analysis

Reversed phase-based liquid chromatography separation was performed using an Elute^®^ UHPLC system with a Bruker Intensity Solo C18 column (100 × 2.1 mm, 1.8 μm) (Bruker Daltonics GmbH, Billerica, MA, USA).

A volume of 5 μL was injected onto the column. The column compartment was heated to 55 °C, while the autosampler was cooled at 8 °C to avoid sample evaporation. Samples were separated with a binary gradient at a constant flow rate of 0.4 mL/min. The mobile phases were composed of solvent A (acetonitrile/water 60:40, 10 mM NH_4_ formate, 0.1% FA) and solvent B (isopropanol/acetonitrile 90:10, 10 mM NH_4_ formate, 0.1% FA). A 20 min gradient was used (0 min 40%B, 1 min 43%B, 1.10 min 50%B, 6 min 54%B, 6.10 min 70%B, 9.0 min 99%B, 20.0 min 99%B). Also, 5 min pre-running was employed to re-equilibrate the column for initial conditions.

The mass spectrometry experiment was undertaken in positive VIP-HESI^®^ mode (Vacuum Insulated Probe Heated Electrospray Ionization) using a timsTOF Pro (Bruker Daltonics GmbH & Co. KG., Billerica, MA, USA) mass spectrometer in PASEF MS/MS mode. The transfer parameters were optimized for mass range (100–1350 *m*/*z*) and mobility range (0.55–1.87 1/K0), and precursors were fragmented from 100 to 1350 *m*/*z*. Mass range was calibrated using the ions of the sodium formate and the ion mobility dimension was calibrated using the ions of the ESI-L Low Concentration Tuning Mix (Agilent Technologies, Santa Clara, CA, USA).

### 4.6. Lipid Annotation

Resulting data were processed considering all four dimensions (*m*/*z*, retention time, ion mobility and intensity) and MS/MS spectra were assigned to them using MetaboScape^®^ 2023b Version 11.0.4 (Bruker Daltonics GmbH & Co. KG., Billerica, MA, USA) in the specified range of 100–1350 *m*/*z*. A T-ReX 4D^®^ algorithm (Bruker Daltonics GmbH & Co. KG., Billerica, MA, USA) was used which combined all adducts and isotopes belonging to the same lipid into features. Feature detection was performed using an intensity threshold of 1500 counts. [M + H]^+^, [M + Na]^+^ and [M + NH_4_]^+^ ions were selected in the ion configuration settings. The processing algorithm combined all adducts and isotopes belonging to the same lipid. 

The features were matched against the open source in silico MS/MS library LipidBlast (Version 68; http://fiehnlab.ucdavis.edu/projects/LipidBlast; accessed on 15 January 2024) [25,39] and rule-based lipid annotation implemented in MetaboScape (algorithm MCube Lipid Species Annotation). The lipid database implemented in this algorithm is structured according to the lipid maps hierarchy (lipid category > lipid main class > lipid sub class). To distinguish lipid species’ accurate *m*/*z* ratiod, isotopic pattern (mSigma value) and CCS values of the precursor, as well as MS/MS spectra, were employed. 

The tentatively assigned lipids are those that received an annotation by rule-based annotation and LipidBlast assignment, both within 2 mDa precursor mass. Additionally, assignments were filtered, matching with scores greater than 600 MSMS, to ensure that lipids were annotated based on high-quality MS/MS spectra and matching with lower than 3% deviations of CCS measured value vs. the predicted CCS value [26,40,41]. The results were manually inspected and potential false positives removed.

The lipid annotations are listed in Supplementary Materials Table S1 and the total number of identified lipids was 131.

### 4.7. Statistical Analysis

Statistical analysis was performed to search the changes in relative abundance of lipids in relation to the scores of the LLPs, eyelid margin alterations and morphological characteristics of the MG. All statistical analyses were performed using Metaboanalys 6.0 (https://www.metaboanalyst.ca; accessed on 15 January 2024). Features with more than 50% missing values were removed. The data were transformed using the base-2 logarithm (log2) to correct for non-normal distributions and standardized by the Autoscaling method (mean-centred divided by standard deviations of each variable) [42].

The 44 samples were classified based on the LLPs, eyelid margin abnormalities (eyelid margin hyperaemia, MG orifice plugging, eyelid margin irregularity and eyelid margin thickening) and morphological characteristics of the MG (MG loss, MG drop out and partial glands) (Supplementary Materials Table S2). Upper and lower eyelid scores were combined for every sample, adding both classifications (Table 2). Samples with mild severity grades for each study characteristic have been excluded from the statistical analysis to examine lipidic differences. Finally, the samples that were included were reclassified into two groups (group 1 and group 2) and the classification scores included in every analysis group are listed in Table 3.

A “supervised orthogonal partial least squares discriminant analysis” (ortho-DA) was conducted to identify lipids with the greatest capabilities to separate the sample groups. Those lipids with VIP from the ortho-DA higher than 1.5 were considered biologically relevant in these analyses. A “hierarchical clustering analysis” (heatmap) was performed based on Euclidean distance and the average cluster algorithm to evaluate changes in the VIP lipid datasets and to separate the samples into groups with lipids of similar relative abundance.

## 5. Conclusions

The study of the lipid profile of the meibum and its relationship with many ocular surface features add valuable information about which lipids enhance or reduce their abundance regarding each sign of alteration. The present study revealed significant variances in lipid abundance between subjects exhibiting no or minimal ocular alterations and those exhibiting higher scores of ocular alterations. The abundance of different lipid species may influence LLPs, eyelid margin hyperaemia, eyelid margin irregularity, MG orifice plugging and MG loss. The current findings have the potential for transfer to the pharmaceutical industry, as novel tear substitutes could be developed and customized for each type of DED. Identifying altered lipids in EDE/MGD could lead to targeted therapies, addressing a critical gap in DED management and potentially benefiting both patients and pharmaceutical industries. The collaboration between basic research and clinical practice showcases how scientific inquiry can enhance patient care. Rapid and cost-effective meibum analysis could revolutionize clinical procedures like routine tests for bodily fluids.

## 6. Future Perspectives

Future research could address several limitations of the current study, such as by extracting meibum from both eyelids separately, or handling a larger sample with different types of DED subjects (EDE/MGD and Aqueous Deficient Dry Eye) at different severity levels. Studying DED types at different severity levels will add valuable information for understanding the DED pathophysiology related to the MGs. In this sense, researchers could find out which lipids are more involved in the chronification of the disease due to their higher or lower abundance on the ocular surfaces at different levels of severity.

## Figures and Tables

**Table 1:** Number of lipids identified according to their lipid class.

Lipid Class		Nº Lipids
Ceramides	(Cer)	15
Hexosyceramides	(HexCer)	1
Monodiacyglycerophosphocholines	(LPC)	8
Diacylglycerophosphocholines	(PC)	15
Diacylglycerophosphoethanolamines	(PE)	2
Diacylglycerophosphoglycerols	(PG)	2
Diacylglycerophosphoinositols	(PI)	1
Ceramide phosphocholenes	(SM)	10
Sphingosines	(SPB)	10
Cholesterol Ester	(CE)	16
Diacyglycerols	(DG)	13
Triacylglycerols	(TG)	38

**Table 2:** Grading scales used to evaluate the ocular parameters related to the meibum.

Procedure		Scheme	Classification Criteria
LLP		Guillon et al. [14]	· Grade 1—Coloured · Grade 2—Amorphous · Grade 3—Fluid · Grade 4—Close Meshwork · Grade 5—Open Meshwork
Eyelid Margin Abnormalities	Hyperaemia	Arita et al. [12]	· Grade 0—No or slight hypaeremia and no telangiectasia · Grade 1—Hyperaemia and no telangiectasia · Grade 2—Hyperaemia and telangiectasia crossing MG in less than half of the full lid margin · Grade 3—Hyperaemia and telangiectasia crossing MG in half or more than half of the full lid margin
	MG orifice plugging	Arita et al. [12]	· Grade 0—No MG orifice plugging · Grade 1—Less than 3 MG plugged · Grade 2—3 or more MG plugged distributed in less than half of the full length of the eyelid margin · Grade 3—3 or more MG plugged distributed in half or more of the full length of the eyelid margin
	Irregularity	Arita et al. [12]	· Grade 0—No irregularity · Grade 1—Less than 3 irregularities · Grade 2—3 or more irregularities
	Eyelid margin thickening	Arita et al. [12]	· Grade 0—No thickening · Grade 1—Thickening with/without rounding · Grade 2—Thickening with diffuse rounding
Meibography	MG loss	Pult et al. [13]	· Grade 1—<25% MG loss · Grade 2—25% to 50% MG loss · Grade 3—50% to 75% MG loss · Grade 4—>75% MG loss
	MG drop out	Arita et al. [12]	· Grade 0—No dropout · Grade 1—Less than 3 dropouts · Grade 2—3 or more dropouts
	Partial glands	Arita et al. [12]	· Grade 0—No partial glands · Grade 1—Less than 3 partial glands · Grade 2—3 or more partial glands and fewer than 3 with loss of half or more of the full MG length · Grade 3—3 or more partial with loss of half or more of the full MG length

LLP: Lipid Layer Pattern; MG: Meibomian Gland.

**Table 3:** Clustering criteria for group 1 and group 2 according to the scores assigned to each characteristic studied.

	Scores Range	Scores Included in Statistical Study		n
		Group 1	Group 2	
LLP	1 to 5	=1	≥4	12
Eyelid Margin Hyperaemia	0 to 6	≤1	≥4	25
MG Orifice Plugging	0 to 6	=0	≥4	12
Eyelid Margin Irregularity	0 to 4	=0	≥3	17
Eyelid Margin Thickening	0 to 4	=0	≥2	36
MG Loss	0 to 6	=0	≥3	23
MG Drop Out	0 to 4	=0	≥3	29
Partial Glands	0 to 6	≤2	≥5	28

Score range: the LLP score is the assigned according to Gillon’s scheme. Eyelid margin abnormalities and morphological features of the MG are expressed as the sum scores for both eyelids in each sample; n: number of samples included in statistical study. LLP: Lipid Layer Pattern; MG: Meibomian Gland.

## Data Availability

The raw data supporting the conclusions of this article will be made available by the authors on request.

## Supplementary Materials

The following supporting information can be downloaded at: https://www.mdpi.com/article/10.3390/ijms25094782/s1.
