# Supplementary material for: Meibum Lipidomic Analysis in Evaporative Dry Eye Subjects
Source: Int J Mol Sci. 2024 Apr 27;25(9):4782. doi: 10.3390/ijms25094782 (PMC11083861; doi:10.3390/ijms25094782)
Supplement: Supplementary file 1 [file ijms-25-04782-s001.zip › ijms-2959352-supplementary.pdf]

International Journal of *Molecular Sciences*

Supplementary material

Table S1. List of lipids identified.

#	Name	m/z meas.	#	Name	m/z meas.	#	Name	m/z meas.
1	CE 16:0	642.61779	45	HexCer 18:1;O2/16:0;O	716.56683	89	SPB 22:0;O2	358.36780
2	CE 18:0	670.64845	46	LPC 16:0	518.32194	90	SPB 22:1;O2	356.35201
3	CE 18:1	668.63326	47	LPC 17:0-SN2	510.35643	91	SPB 24:1;2O	384.38349
4	CE 18:2	666.61731	48	LPC 18:0	524.37067	92	SPB 26:1;2O	412.41458
5	CE 20:0	698.68031	49	LPC 18:1	522.35503	93	SPB 28:1;2O	440.44600
6	CE 20:1	696.66490	50	LPC 18:1-SN2	522.35506	94	TG 10:0_10:0_18:1	682.59821
7	CE 20:2	694.64854	51	LPC 18:2	542.32069	95	TG 10:0_18:2_18:2	788.67609
8	CE 20:3	692.63231	52	LPC 18:2-SN2	520.33932	96	TG 12:0_12:0_18:1	738.65987
9	CE 22:0	726.71054	53	LPC 18:3	518.32348	97	TG 12:0_14:0_16:0	740.67584
10	CE 22:1	724.69596	54	PC 12:0_24:4	782.56721	98	TG 14:0_16:1_16:1	792.70603
11	CE 22:2	722.68013	55	PC 16:0_18:1	760.58498	99	TG 14:1_16:0_16:1	792.70720
12	CE 22:3	720.66402	56	PC 25:0_13:1	816.64689	100	TG 14:1_16:1_16:1	790.69107
13	CE 24:0	754.74222	57	PC 34:2	758.56917	101	TG 14:1_16:1_18:1	818.72412
14	CE 24:2	750.71262	58	PC 34:3	756.55299	102	TG 14:1_16:1_18:2	816.70674
15	CE 24:3	748.69546	59	PC 36:2	786.59924	103	TG 15:0_16:1_16:1	806.72265
16	CE 25:0	768.75799	60	PC 36:3	784.58562	104	TG 16:0_16:1_18:1	848.76939
17	Cer 10:1;2O/28:7	580.47271	61	PC 38:6	806.56875	105	TG 16:0_16:1_18:2	846.75398
18	Cer 16:1;O2/22:0	594.58172	62	PC 42:2	870.69369	106	TG 16:0_18:0_18:1	878.81781
19	Cer 16:1;O3/20:0;O	598.53976	63	PC O-16:0	496.33962	107	TG 16:0_18:0_20:0	908.86270
20	Cer 17:0;3O/23:0;(2OH)	656.61755	64	PC O-18:0	524.37042	108	TG 16:0_18:1_18:1	876.80029
21	Cer 18:0;O2/16:0	540.53541	65	PC O-18:1	522.35520	109	TG 16:1/16:1/16:1	818.72301
22	Cer 18:1;2O/17:0	552.53474	66	PC O-20:4	544.33958	110	TG 16:1_16:1_18:1	846.75433
23	Cer 18:1;O2/16:0	538.51887	67	PC O-36:1	774.63792	111	TG 16:1_17:1_17:1	846.75413
24	Cer 18:1;O3/20:0;O	626.57075	68	PC O-36:2	772.62063	112	TG 16:1_17:1_18:1	860.76978
25	Cer 18:2;O2/16:0	536.50321	69	PE 36:1	746.57042	113	TG 16:1_18:1_18:1	874.78702
26	Cer 19:0;2O/17:2	564.53485	70	PE 36:2	744.55386	114	TG 16:1_18:1_20:1	902.81661
27	Cer 19:0;2O/18:2	578.55056	71	PG 18:1/18:1	792.57411	115	TG 17:0_18:1_18:1	890.81769
28	Cer 19:0;2O/19:2	592.56543	72	PG 18:1_20:3	816.57395	116	TG 17:1_18:1_18:1	888.80164
29	Cer 19:0;3O/17:0;(2OH)	600.55552	73	PI 38:4	904.59121	117	TG 18:0_18:1_18:2	902.81594
30	Cer 23:0;3O/16:0;(2OH)	642.60216	74	SM 30:1;2O/10:0	787.66865	118	TG 18:0_22:0_22:0	1020.98920
31	Cer 34:2;2O/2:0	564.53575	75	SM 32:1;O2	675.54215	119	TG 18:1_18:1_19:1	916.83227
32	DG 14:0_16:0	558.50797	76	SM 34:1;2O/6:0	787.66890	120	TG 18:1_18:1_20:0	932.86403
33	DG 14:0_18:1	584.52458	77	SM 34:1;O2	703.57503	121	TG 18:1_18:1_20:1	930.84925
34	DG 16:1/16:1	582.50913	78	SM 34:1;O3	719.57032	122	TG 18:1_18:1_20:3	926.81572
35	DG 16:1_17:1	596.52407	79	SM 42:1;O3	831.69454	123	TG 18:1_18:1_25:0	1002.94099
36	DG 16:1_18:1	610.53999	80	SM 44:0;3O	861.74110	124	TG 18:1_20:3_20:3	950.81649

37	DG 16:1_18:2	608.52618	81	SM 44:0;O2	845.74552	125	TG 8:0_10:0_15:0	614.53480
38	DG 18:1/18:1	638.57153	82	SM 44:1;O2	843.73203	126	TG 8:0_12:0_12:0	600.51968
39	DG 18:1_18:2	636.55594	83	SM 45:0;3O	875.75744	127	TG 8:0_18:1_18:1	764.67536
40	DG 18:1_20:3	662.57186	84	SPB 18:0;3O	318.30001	128	TG 8:0_18:1_18:2	762.65979
41	DG 18:2/18:2	634.54025	85	SPB 19:0;3O	332.31586	129	TG 8:0_8:0_10:0	516.42566
42	DG O-19:0_16:1	612.59151	86	SPB 20:0;2O	330.33652	130	TG 8:0_8:0_16:1	598.50405
43	DG O-23:0_18:1	696.68522	87	SPB 20:0;O3	346.33141	131	TG 9:0_9:0_9:0	530.44130
44	DG O-38:5	646.57755	88	SPB 21:0;2O	344.35174			

Table S2. Total scores for each characteristic studied. The LLP score is the assigned according to Gillon's scheme. Eyelid margin abnormalities and morphological features of the MG, such as hyperemia, MG orifice plugging, irregularity, MG loss, MG drop out and partial glands, are expressed as the sum scores for both eyelids in each sample. ID: identity; LLP: Lipid Layer Pattern; MG: Meibomian Gland.

Sample ID	LLP	Eyelid Margin Hyperemia	MG Orifice Plugging	Eyelid Margin Irregularity	Eyelid Margin Thickening	MG Loss	MG Drop Out	Partial Glands
1	5	5	4	1	2	2	1	4
2	4	4	4	1	0	2	2	4
3	4	2	1	2	2	3	1	6
4	4	4	3	4	4	3	0	5
5	3	5	4	3	1	3	0	5
6	3	4	3	3	2	4	1	5
7	4	5	0	2	2	1	0	2
8	3	4	1	1	0	0	1	2
9	4	1	1	0	0	0	0	2
10	5	3	1	0	0	0	0	2
11	4	1	0	0	0	3	1	3
12	4	1	1	0	0	3	1	4
13	4	5	4	1	3	4	3	6
14	3	5	2	2	2	5	3	6
15	5	4	1	0	0	3	1	5
16	4	5	1	1	0	2	1	4
17	4	3	6	1	0	3	4	6
18	5	5	6	2	0	5	4	6
19	5	5	2	2	2	2	0	3
20	5	4	2	2	1	3	0	3
21	4	4	2	0	0	3	0	5
22	5	4	2	1	1	2	0	4
23	4	3	2	0	0	2	0	1
24	4	3	1	1	0	1	0	1
25	4	5	0	1	2	2	0	5

26	4	3	2	1	2	2	0	4
27	4	0	0	0	0	2	2	3
28	4	0	0	0	0	1	2	2
29	1	2	2	0	0	0	1	2
30	3	3	2	2	2	1	0	3
31	4	3	1	1	2	1	0	3
32	5	2	1	2	1	2	0	4
33	3	2	1	1	0	2	0	4
34	2	3	1	0	0	2	0	2
35	2	3	3	0	2	2	0	2
36	4	5	2	1	2	0	1	2
37	4	5	2	1	1	0	0	0
38	4	2	1	1	0	0	0	0
39	4	3	2	1	1	1	1	0
40	4	4	2	0	0	3	0	4
41	4	3	2	0	0	2	0	3
42	4	4	0	1	0	1	0	2
43	2	4	3	2	2	5	3	6
44	4	5	1	1	0	3	2	4
